# Association between circadian rhythm disturbances and cognitive decline in the elderly: a systematic review

**DOI:** 10.1007/s10072-026-09221-y

**Published:** 2026-07-16

**Authors:** Letícia Guimarães Lopes, Adriane Santos Oliveira, Camila Carvalho Fingergut, Gabriela Nunes E Brito, João Victor Pereira Gonzalez, Kenzo Ogasawara Donato, Miguel Meira e Cruz, Cristina Salles

**Affiliations:** 1 Department of Medicine, Bahiana School of Medicine and Public Health, Av. Dom JoãoVI, 275, Brotas, Salvador, Bahia 40290-000 Brazil; 2 Department of Sleep Medicine, Lisbon School of Medicine, Cardiovascular Center of Lisbon University, Lisbon, Portugal

**Keywords:** Circadian rhythm disorders, Cognitive decline, Elderly, Mild Cognitive Impairment, Circadian dysregulation

## Abstract

**Background:**

Circadian rhythm dysregulation may contribute to sleep-wake disorders and cognitive impairment. In the elderly, circadian abnormalities have been observed in Mild Cognitive Impairment (MCI), suggesting a possible association between neurocognitive deficits and circadian dysfunctions.

**Objective:**

To analyze the association between circadian rhythm disturbances and cognitive decline in the population aged ≥ 60 years.

**Methods:**

This systematic review included studies evaluating the association between circadian rhythm disturbances and cognitive decline in elderly individuals aged 60 years or older, with self-reported or objectively measured sleep data and cognitive assessment via questionnaires. Studies tracking progression from MCI to Alzheimer’s disease (AD) were retained, as they capture circadian changes across the MCI-to-dementia continuum. Studies that evaluated other sleep disorders and/or other conditions of cognitive impairment exclusively or that did not specify the mean age of the participants were excluded.

**Results:**

Ten studies were included, totaling 5,731 participants *(5*,*707 eligible for analysis*). Circadian exposures were organized into three domains: (1) actigraphic rest-activity rhythm metrics (amplitude, robustness, interdaily stability, intradaily variability, and acrophase); (2) sleep quality and architecture parameters (efficiency, fragmentation, latency, WASO, and melatonin timing); and (3) circadian-disrupting lifestyle exposures (rotating night shift work). Across these domains, circadian dysregulation was consistently associated with a higher risk of development or progression of MCI and dementia.

**Conclusion:**

Circadian and sleep disturbances negatively impact cognitive health in the elderly, reinforcing the need for further research on this association and its public health implications.

## Introduction

Circadian rhythm disturbance, whether primary or secondary, can arise from recurrent disruption of the ideal timing of sleep and wakefulness. From this, the patient is at risk of developing a sleep disorder, usually associated with sleepiness or insomnia, that misaligns the individual’s circadian sleep-wake pattern with that of the environment, thereby affecting social, professional, and other relevant areas of the patient’s life [1].

The clinical relevance of these disorders is even more pronounced in the context of aging, a moment when the regulatory mechanisms of the circadian rhythm undergo physiological changes, including reduced amplitude of biological rhythms, nocturnal sleep fragmentation, and decreased nocturnal melatonin secretion. However, the severity of these changes becomes evident when they compromise essential biological functions and negatively impact mental health, the immune system, and, above all, cognition [2, 3].

Studies indicate that circadian dysfunctions—such as delay or advance in melatonin release, nocturnal sleep fragmentation, and reduced amplitude of biological rhythms—may be associated with changes in critical brain regions, such as the hippocampus, the prefrontal cortex, and structures directly involved in memory consolidation and executive functions, especially in Mild Cognitive Impairment (MCI) [4, 5]. This condition is often considered an intermediate stage between normal aging and dementia, and commonly impacts the patients’ quality of life in areas such as autonomy, social participation, and the performance of various activities, especially complex ones [6, 7].

Changes in circadian rhythms that interfere with the rest-activity cycle, sleep quality, body temperature, and melatonin secretion are recurrently observed in elderly individuals with mild cognitive impairment. Evidence indicates that up to 36.9% of the elderly with multiple comorbidities present with MCI, with poor sleep quality being a factor of strong correlation [8]. High scores on the Pittsburgh Sleep Quality Index (PSQI) have been associated with an increased risk of MCI [3], suggesting not only preventive interventions targeting neurocognitive dysfunction in this population but also a possible association between the two clinical conditions. However, the exact mechanisms by which circadian rhythm disturbances contribute to the development and/or progression of mild cognitive impairment in the elderly, as well as the modulating factors of this relationship, have not been sufficiently explored in previous studies.

Existing reviews have addressed specific aspects of this relationship — such as circadian changes in healthy aging and MCI, objective sleep measures in MCI, and broader links between sleep disturbance and cognitive decline [3]. However, no prior synthesis has systematically integrated circadian exposures across the three domains that the available evidence spans: (1) actigraphic rest-activity rhythm parameters; (2) sleep quality and architecture, including melatonin timing; and (3) circadian-disrupting lifestyle factors such as rotating night shift work. Rather than proposing an entirely new conceptual framework, the present review’s contribution lies primarily in this cross-domain synthesis: it integrates evidence across these three domains and explicitly evaluates studies that track the MCI-to-AD continuum, an approach that, to our knowledge, has not been previously undertaken in a single systematic review.

Thus, this systematic review aims to synthesize the evidence available on the nature and strength of the possible association existing between circadian rhythm disturbances and the development of mild cognitive impairment in the elderly, updating the understanding of this complex relationship and identifying gaps for future investigations.

## Methods

### Study design and adherence to guidelines

This systematic literature review was conducted in accordance with the Preferred Reporting Items for Systematic Reviews and Meta-Analyses (PRISMA 2020) guidelines. The protocol for this review was prospectively registered in PROSPERO (International Prospective Register of Systematic Reviews) under the ID: CRD42024564508, ensuring transparency and preventing publication bias.

### Search strategy

A comprehensive search strategy was performed and applied in the PubMed, Cochrane Library, and Embase databases, covering the period from their inception in July 2024 until January 2025, without time restrictions. The descriptors indexed in the Medical Subject Headings (MeSH) and Health Sciences Descriptors (DeCS) were combined by Boolean operators (‘AND’ and ‘OR’). Among the search terms, the following were included: “Circadian Rhythm”, “Cognitive Impairment”, and “elderly”, obtaining the following search detail: (Circadian Rhythm OR Chronobiology OR “Shift Work”) AND (“Cognitive impairment” OR Dementia OR “Cognitive dysfunction”) AND (aged OR elderly OR Older). The searches were complemented by a manual search for the references listed in the included articles, with the focus of improving the work by identifying additional relevant studies. To ensure the currency of the evidence base prior to publication, the same search strategy was re-run in June 2026 across the three databases; no additional studies meeting the pre-defined eligibility criteria were identified beyond those already retrieved by the original January 2025 search (which had included, among others, Haghayegh et al. and Milton et al., discussed in the Discussion as non-eligible contextual evidence).

### Eligibility criteria

**Inclusion criteria**: (1) Population: Elderly individuals (mean age > = 60 years) with or at risk of mild cognitive impairment; (2) Exposure: Studies that investigated the association or impact of circadian rhythm disturbances; (3) Outcomes: Studies that presented data on the association between circadian rhythm disturbances and cognitive decline, including self-reported and/or objectively measured sleep data and cognitive assessment, mainly through validated questionnaires; (4) Study Design: Observational studies.

**Exclusion criteria**: Studies that evaluated exclusively other sleep disorders and/or other conditions of cognitive impairment (e.g., advanced dementia, primary psychiatric disorders); Studies that did not present clear data on the mean age of the participants or that included a predominantly non-elderly population in the analysis; Review articles, case reports, editorials, case series, letters, conference abstracts, systematic reviews, and meta-analyses. Studies in which Alzheimer's disease appeared as a progression endpoint from a baseline MCI or cognitively healthy population were not excluded, as they allow the characterization of circadian disturbances across the MCI-to-dementia trajectory. Only studies that enrolled exclusively patients with established advanced dementia, without an MCI or healthy-aging comparator, were excluded.

### Identification and selection of studies

Initially, the process of selecting the articles was carried out by two independent authors, based on the eligibility criteria. This primary evaluation was based on the title and abstract, followed by reading the pre-selected articles in full. At the end of the selection, a third author was responsible for resolving cases of divergence after discussion with the others. A manual screening was performed on the references of the included articles, following the same criteria. The authors used the systematic review management application – Rayyan [9] – developed by the Qatar Computing Research Institute to assist in the selection of articles.

### Data extraction

From the articles included in the review, the following data were extracted in a standardized form: title; author(s); year of publication; country of origin; study design; period of activity; sample size; diagnostic criteria for mild cognitive impairment; subtypes of mild cognitive impairment; assessment questionnaires for mild cognitive impairment; assessment questionnaires for sleep and/or circadian rhythm disturbance; circadian rhythm disturbance; cortisol, memory, and temperature analysis; mean age of participants; gender of participants; and conclusions. Data extraction was performed by two independent reviewers using a standardized spreadsheet. Any divergences were resolved by consensus or with the intervention of a third researcher.

### Outcomes

The outcomes of interest for this review were defined as:

#### Primary outcome

Impact of circadian rhythm disturbances on the incidence and/or progression of Mild Cognitive Impairment (MCI) in the elderly: Evaluated by the correlation or association between the presence of circadian disturbances, defined by changes in: phase advance/delay, sleep fragmentation, total nocturnal sleep duration, sleep latency, sleep efficiency, wake after sleep onset (WASO); and the diagnosis or worsening of MCI, defined by Petersen’s criteria, neuropsychological assessment, and scores in specified cognitive screening tests.

#### Secondary outcomes

Differences in circadian patterns between the control group and individuals with MCI: Comparison of circadian parameters (rest-activity rhythm amplitude, melatonin peak time) between elderly individuals with MCI and cognitively healthy controls.

Evaluation of cognitive performance in the elderly with circadian rhythm disturbances: Analysis of scores on specific cognitive tests (memory, executive functions) in the elderly with and without circadian disturbances.

Evaluation of sleep patterns and biological markers: Analysis of sleep data, such as sleep latency, sleep efficiency, PSQI scores, among others, in relation to MCI.

### Quality and risk of bias assessment

The methodological quality and risk of bias of the included studies were evaluated independently by two reviewers. For observational studies, the Strengthening the Reporting of Observational Studies in Epidemiology (STROBE) [10] was used as a parameter for evaluating the quality of the article, classifying each item as ‘fully met’, ‘partially met’, or ‘not clear’. Additionally, the criteria of the Risk Of Bias In Non-randomized Studies – of Exposures (ROBINS-E) [11] tool were employed to assess risk of bias across seven domains (no information, low, moderate, serious, or critical). Any disagreements between the reviewers were resolved by consensus or, when necessary, with the mediation of a third researcher.

## Results

**Fig. 1:**
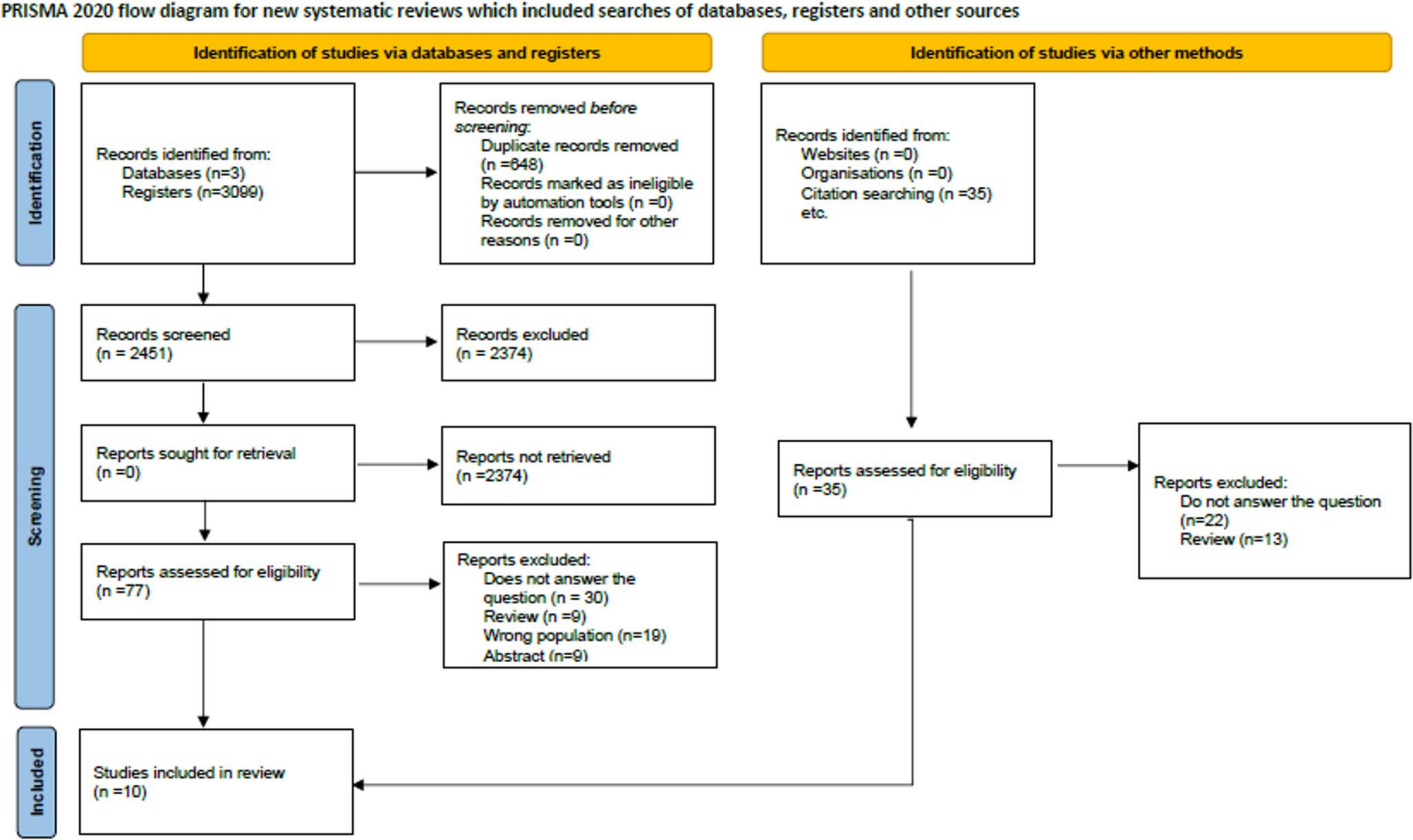
Flow diagram of the identification, screening, eligibility, and inclusion of studies in the systematic review (PRISMA 2020)

After the searches in the PubMed, Cochrane Library, and Embase databases, a total of 3,099 articles were identified. Among these studies, 648 were excluded due to duplication, and 2,374 were excluded for not meeting the eligibility criteria. After a complete review of the remaining 77 studies, 67 were eliminated due to inadequacies regarding inclusion and exclusion criteria. An additional manual search of the references of the 10 included articles (*n* = 35) was performed, but did not result in the inclusion of more studies (Fig. 1).

### Quality and risk of bias analysis

**Fig. 2:**
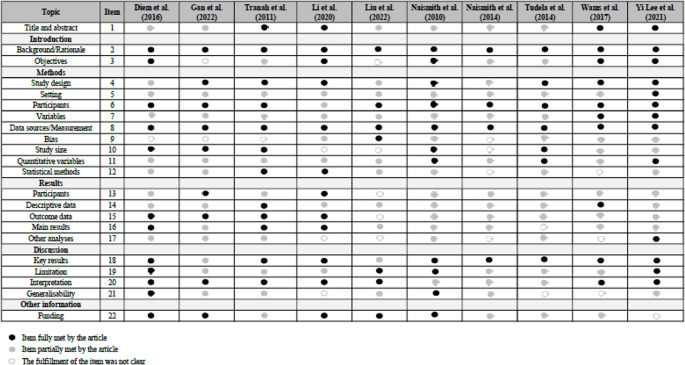
Quality assessment of observational studies using the STROBE protocol

The STROBE protocol evaluated the quality of the observational studies. As evidenced in (Fig. 2), none of the articles fully met all the suggested items, with Yi Lee et al. [12] being the one that best adhered to the protocol guidelines. The study by Naismith et al. [13] obtained thelowest score.

**Fig. 3:**
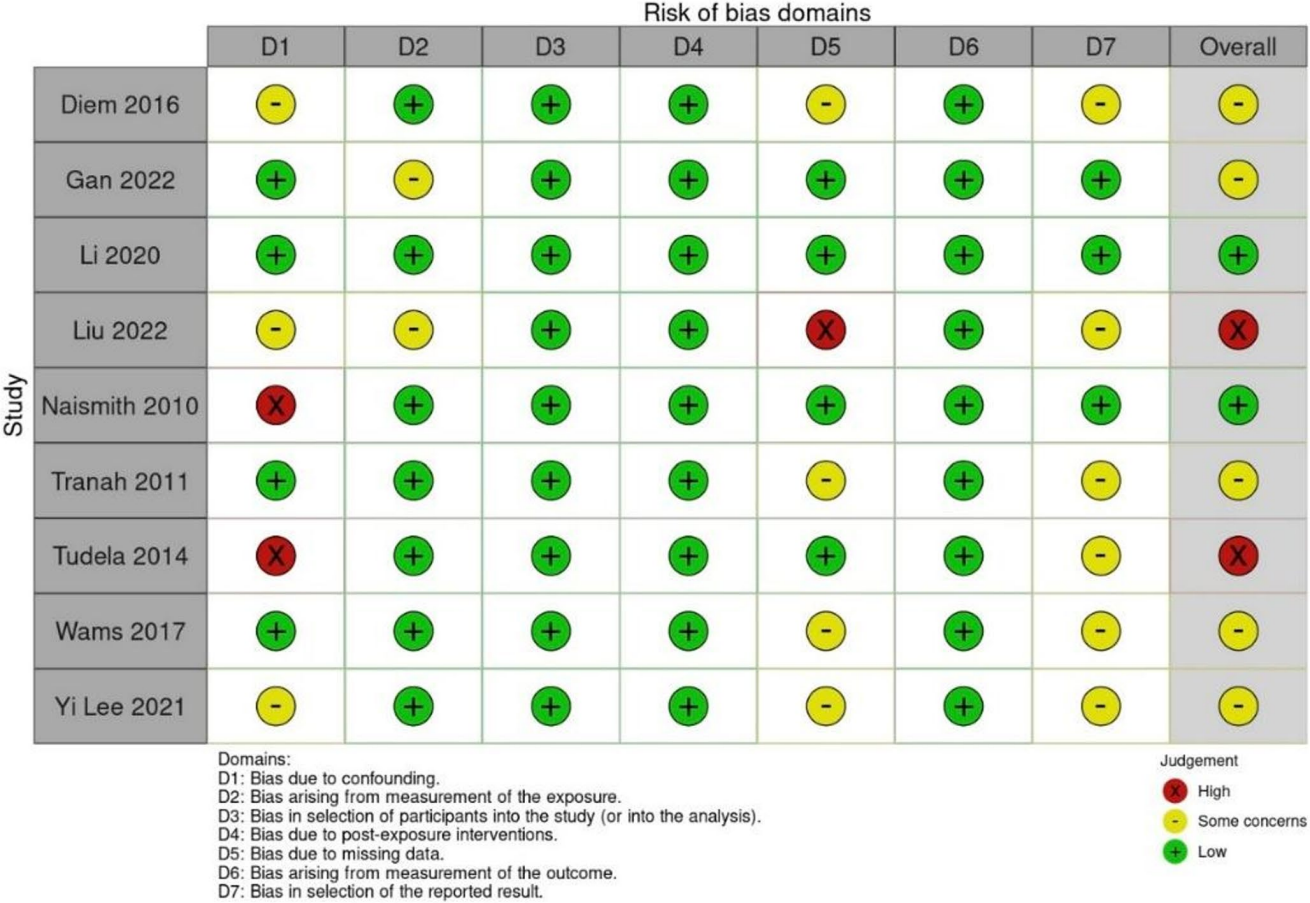
Risk of bias assessment of non-randomized studies using the ROBINS-E tool

**Fig. 4:**
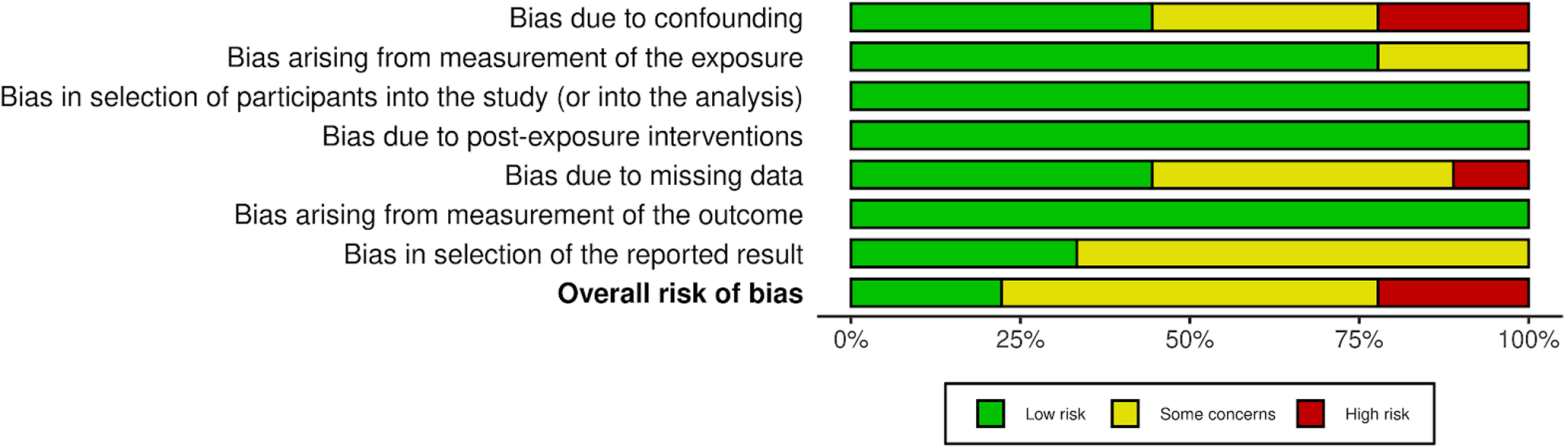
ROBINS-E tool summary

The risk of bias was evaluated using the ROBINS-E tool. As shown in (Fig. 3), two studies (Liu et al. [14] and Ortiz-Tudela et al. [15]) were considered to be at high general risk, mainly due to missing data (D5) and confounding factors (D1), respectively. Most of the other studies were classified as low risk or with some concerns, particularly regarding confounding factors (D1), missing data (D5), and selective reporting (D7), while the exposure and outcome measurements (D2, D6) presented, in general, low risk. The distribution at the domain level is summarized in (Figs. 3 and 4).

### Participant´s characteristics

The main characteristics of the participants and the included studies are reported in Table 1.

**Table 1:** General sample characteristics

Author (year)	Country	Study Design	Sample (*n*)	MCI/Control (*n*)	Mean age (years)		Men/ Women
					MCI	Control	
Diem et al. (2016)[16]	United States	Prospective cohort	1245	290/772	82.63 ± 3.32ª		0/1245
Gan et al. (2022)[17]	China	Cross-sectional	1576	920/650ᵇ	65,4 ± 8	65 ± 5,2	0/1576
Tranah et al. (2011)[18]	United States	Prospective cohort	1282	302/785	83,0ᶜ		0/1282
Li et al. (2020)[19]	United States	Prospective cohort	1203	NR	81.8 ± 3.7ᵈ		274/929
Liu et al. (2022)[14]	China	Prospective cohort	99	68/31	67 ± 9	66 ± 6	39 /60
Naismith et al. (2010)[13]	Australia	Prospective cohort	15	15/0	66,7	NR	10/5
Naismith et al. (2014)[20]	Australia	Cross-sectional	52	26/26	70,1 ± 9,9	65,9 ± 9,8	29/23
Ortiz-Tudela et al. (2014)[15]	Spain	Cross-sectional	40	21/19	74,1 ± 1.5	71,7 ± 1,4	11/29
Wams et al. (2017)[21]	United Kingdom	Cross-sectional	45	8/13ᵉ	77,1 ± 4.0	73,8 ± 4,6	25/20^*e*^
Yi Lee et al. (2021)[12]	Hong Kong	Prospective cohort	174	51/123ᶠ	77,5 ± 7,1	74,9 ± 6,8	36/138

ª Mean age calculated based on information provided in the study.

ᵇ Although MCI incidence was assessed in both groups, the original study divided participants in this manner.

ᶜ Study reported mean age for four quartiles but did not specify the number of participants or mean age for dementia, MCI, or healthy subgroups; therefore, a specific subgroup calculation was not possible, and the overall mean age from the abstract was used.

ᵈ Study analyzes the 15-year progression from cognitive health to MCI and subsequently to Alzheimer’s disease; the cohort was cognitively healthy at baseline.

ᵉ The original study by Wams et al. enrolled 45 participants in total; twenty-four were diagnosed with Alzheimer’s disease and did not meet this review’s inclusion criteria. Only the remaining 21 participants (aMCI n = 8; cognitively healthy controls n = 13) were extracted and analyzed in this review; the sample size of n = 45 shown in this table, as well as the Men/Women count of 25/20, reflect the original study's total enrollment (including the 24 excluded AD patients), not the analyzed subsample. The original publication did not report sex distribution separately for the aMCI and control subgroups, so a corrected breakdown could not be provided.

ᶠ Sixteen of the 51 participants presented cognitive decline and were grouped with MCI-diagnosed individuals for analysis.

### Domain 1: actigraphic rest-activity rhythm metrics

Four prospective cohort studies employed actigraphy to quantify rest-activity circadian parameters — amplitude, robustness, interdaily stability (IS), intradaily variability (IV), and acrophase — and examined their association with incident MCI or dementia.

Tranah et al. (2011) [18], in a large sample of community-dwelling older women (n = 1,282; mean age 83 years), demonstrated that lower rest-activity amplitude, reduced rhythm robustness, and a delayed acrophase were significant prospective predictors of incident dementia and MCI over five years. The study by Li et al. (2020) [19] extended this finding to the MCI-to-AD continuum: in a cohort of 1,203 participants followed for an average of 5.8 years, lower circadian amplitude, decreased IS, and increased IV were consistently associated with a higher risk of progression to Alzheimer's disease — both in participants with normal cognition at baseline and in those with MCI.

Yi Lee et al. (2021) [12], in a population-based prospective study in Hong Kong (n = 174; mean age 74.9–77.5 years), demonstrated that a delayed rest-activity acrophase was associated with a significantly lower probability of maintaining cognitive function over 12 months, further supporting a temporal link between acrophase displacement and cognitive trajectories. Diem et al. (2016) [16], using the same Study of Osteoporotic Fractures database as Tranah et al. [18], reported that lower sleep efficiency and longer sleep latency — actigraphically derived — were independently associated with a higher risk of MCI or dementia in women followed for approximately five years.

### Domain 2: sleep quality and architecture

Several included studies evaluated circadian disruption through sleep quality indices, detailed polysomnographic or actigraphic sleep architecture, and neuroendocrine markers such as melatonin.

Naismith et al. (2010) [13] observed that individuals with non-amnestic MCI (naMCI; *n* = 15; mean age 66.7 years) displayed fragmented sleep, characterized by prolonged rest periods and increased wake after sleep onset (WASO), which correlated with deficits in attention, non-verbal learning, and executive functions, independent of depressive symptoms. In a subsequent study, Naismith et al. (2014) [20] enrolled 52 participants with MCI and age-matched controls (mean age 70.1 vs. 65.9 years), measuring salivary melatonin to determine Dim Light Melatonin Onset (DLMO) alongside polysomnography. Participants with MCI showed an earlier DLMO (phase advance), greater WASO, and longer REM latency, with the phase advance correlating with poorer overnight memory consolidation. These findings underscore that circadian disruption in MCI extends beyond actigraphy-measurable activity patterns to hormonal timing and sleep microarchitecture.

Liu et al. (2022) [14], in a Chinese cross-sectional study (*n* = 99; MCI *n* = 68), used a wrist-worn sensor and the PSQI to characterize both subjective sleep quality and objective circadian autonomic variability. Participants with MCI exhibited a blunted nocturnal peak in heart rate variability, decreased autonomic oscillation amplitude, and greater nocturnal oxygen desaturation, alongside reduced morning physical activity.

Ortiz-Tudela et al. (2014) [15], employing the composite Circadian Function Index (CFI — integrating relative amplitude, intradaily variability, and interdaily stability), found that patients with multi-domain MCI showed a phase advance in wrist temperature, motor activity, and body position rhythms compared to healthy controls. Wams et al. (2017) [21] (aMCI *n* = 8, controls *n* = 13; mean age 77.1 vs. 73.8 years) found that, despite similar circadian amplitude to controls, participants with amnestic MCI (aMCI) showed significant impairment in working and verbal memory tasks — approaching deficits seen in Alzheimer’s disease patients — while sleep quality and quantity remained comparable to controls. The original study by Wams et al. also enrolled a separate group of patients with established Alzheimer’s disease (*n* = 24); these AD patients were not included in our data extraction or analysis, as they did not meet this review’s eligibility criteria for advanced dementia. Only the aMCI and cognitively healthy control groups from this study were analyzed (see Table 1, footnote e).

### Domain 3: circadian-disrupting lifestyle exposures

Gan et al. (2022) [17] examined the effect of a socially imposed circadian disruptor — rotating night shift work (RNSW) — in a cross-sectional study of 1,576 retired nurses (920 MCI / 650 controls; mean age 65.4 years). Retired nurses with a history of RNSW showed a significantly higher prevalence of both amnestic and non-amnestic MCI, as well as dementia, compared to retired teachers. The association was particularly strong in individuals over 70 years, and daytime napping did not compensate for the cognitive effects of chronic circadian misalignment.

It is important to note that the three domains above do not represent equivalent markers of circadian dysregulation. Domain 1 comprises direct circadian markers obtained through actigraphy and chronobiological modeling (amplitude, robustness, interdaily stability, intradaily variability, and acrophase). Domain 2 combines a smaller subset of direct circadian markers (melatonin timing/DLMO, wrist temperature rhythm, and the composite Circadian Function Index) with sleep-continuity measures that index sleep quality and architecture rather than circadian phase or amplitude per se (sleep efficiency, fragmentation, latency, and WASO), as well as autonomic and oxygenation parameters that are physiologically downstream of, but not direct readouts of, the central circadian oscillator. Domain 3 comprises an indirect, lifestyle-based proxy for circadian disruption (rotating night shift work) rather than a measured circadian parameter. This distinction is maintained throughout the Results and Discussion to avoid treating heterogeneous measures as interchangeable.

Table 2 summarizes the main assessment mechanisms for MCI and sleep/circadian rhythm used in the articles; Table 3 presents the values obtained by their respective authors regarding objective sleep pattern measurement data; and Table 4 highlights the main outcomes identified in the articles concerning the association between circadian rhythm disorders and mild cognitive impairment in the elderly.

**Table 2:** Assessment mechanisms for MCI and sleep/circadian rhythm

Author (year)	Cognitive Assessment Questionnaire	MCI Subtypes	Sleep/Circadian Rhythm Analysis	Key Sleep-Wake Parameters Evaluated
*Diem et al. (2016)*[16]	3MS; CVLT; IQCODE; GDS	NR	Actigraphy; Sleep diaries	Total sleep time per night, sleep latency, and efficiency, WASO
*Gan et al. (2022)*[13]	MMSE; MoCA; ADL.	aMCI; naMCI; multiple or single domain	Rotating night shift work (RNSW) and daytime sleep after night shift (Daytime Recovery - DTR)	RNSW duration in years; frequency (number of night shifts per month); duration of DTR
*Tranah et al. (2011)*[18]	3MS; CVLT; IQCODE; GDS	NR	Actigraphy	Acrophase; rhythm amplitude; robustness
*Li et al.* *(2020)*[19]	MMSE	NR	Actigraphy	Acrophase; rhythm amplitude; robustness; IS; IV; total nocturnal sleep duration; sleep fragmentation index
*Liu et al.* *(2022)*[14]	MoCA; AD8; IADL; GDS-15	NR	PSQI; wrist-worn triaxial accelerometer and optical heart rate (HR) sensor	Circadian rhythm variability, including HR variation and oxygen saturation
*Naismith et al. (2010)*[13]	MMSE; WAIS-III; WTAR	naMCI	Wrist actigraphy; sleep diaries; self-report forms; PSQI; Horne-Östberg MEQ	Onset, offset, total time, and rest efficiency; sleep latency; total sleep time; number and average duration of awakenings; WASO
*Naismith et al. (2014)*[20]	MMSE	aMCI, naMCI	Actigraphy; sleep diaries; salivary melatonin; PSQI; ESS; MEQ; Polysomnography	Onset, offset, and total sleep duration; time spent in NREM and REM sleep; REM latency; WASO; DLMO
*Tudela et al. (2014)*[15]	MMSE; TAVEC; DS	aMCI, naMCI, multidomain	Wrist temperature rhythm; CFI; actimeter; light meter	Pulse temperature onset; IV; IS; RA; body position and rest-activity rhythm; ambient light exposure
*Wams et al. (2017)*[21]	MMSE; ADL.	aMCI	Wrist actigraphy; sleep diaries; PSQI; JSQ	Period duration, IV, IS, activity in the 5 least active hours and 10 most active hours, number of wake periods, fragmentation index, time in bed, total time, efficiency, sleep onset, and offset
*Yi Lee et al. (2021) *[12]	MoCA	NR	Actigraphy	MESOR, amplitude, acrophase, percentage rhythm

*3MS* Modified Mini-Mental State Examination, *AD8* Ascertain Dementia 8-item Questionnaire, *ADL* Activity of Daily Living Scale, *CFI* Circadian Function Index, *CVLT* California Verbal Learning Test, *DLMO* Dim light melatonin onset, *DS* Blessed Dementia Scale, *ESS* Epworth Sleepiness Scale, *GDS-15* Geriatric Depression Scale-15, *IADLs* Instrumental Activities of Daily Living Scale, *IQCODE* Informant Questionnaire on Cognitive Decline in the Elderly, *IS* Interday Stability, *IV* Intraday Variability, *JSQ* Jupiter Medical Center Sleep Questionnaire, *MEQ* Morningness-Eveningness Questionnaire, *MMSE* Mini-Mental State Examination, *MoCA* Montreal Cognitive Assessment, *PSQI* Pittsburgh Sleep Quality Index, *RA* Relative Amplitude, *TAVEC* Test from Complutense University, *WAIS-III* Wechsler Adult Intelligence Scale Third Edition, *WTAR* Wechsler Test of Adult Reading.

**Table 3:** Objective sleep measurement data for patients with MCI / Dementia

Author	WASO (minutes)	Acrophase (hour)	Rhythm Amplitude (counts/minute)	Total Sleep time (hours/minutes)	Sleep Latency (minutes)	Sleep Efficiency (%)
*Diem et al. (2016)*[16]ª	58.3 (39.3–85.8)	NR	NR	6.91 (6.11–7.51)	28.8 (17.3–47.5)	81.8 (74.4–86.3)
*Tranah et al. (2011)*[18]ᵇ	NR	1:34 − 3:51	≥ 4194	405.51 (63.24) (*n* = 322) 410.19 (63.01) (*n* = 322) 410.42 (64.39) (*n* = 318) 406.14 (85.64) (*n* = 320)	NR	80.11 (9.01) (*n* = 322) 79.89 (9.35) (*n* = 322) 79.63 (9.91) (*n* = 318) 76.49 (13.64) (*n* = 320)
*Li et al. (2020)*[19]	NR	13·20 (1·78)	0·32 (0·11)ᶜ	5·61 (1·45)	NR	NR
*Naismith et al. (2010)*[13]	61.78 (15.5)	NR	NR	518.00 (165.3)ᵈ	28.93 (45.6)ᵈ	61.8 (0.2)
*Naismith et al. (2014)*[20]	124.6 (62.3)	NR	NR	381.5 (82.1)	121.4 (89.9) REM sleep	NR
*Wams et al. (2017)*[21]ᵉ	NR	NR	0.913 (0.047)	7.015 (0.38)	NR	90.9 (3.5)
*Yi Lee et al. (2021)*[12]	NR	< 1:24: (*n* = 19) 1:24 − 3:00: (*n* = 14) 3:00: (*n* = 18)	≤ 109.60: (*n* = 17) 109.60-168.78: (*n* = 17) 168.78: (*n* = 17)	NR	NR	NR

ª Values represent the median figures provided in the study, as the cohort was subdivided into four amplitude quartiles.

ᵇ Overall mean amplitude for the study population was not explicitly reported; the values entered in the table are those used as reference within the study population.

ᶜ Values expressed in “normalized units”, referring to the activity amplitude (difference between peak and trough of the circadian cycle) adjusted by standard deviation.

ᵈ Total sleep time and sleep latency values (in minutes) were recorded via sleep diaries rather than actigraphy, and thus represent subjective data within the respective study.

ᵉ This value refers specifically to patients with amnestic Mild Cognitive Impairment (aMCI) and does not include those diagnosed with Alzheimer’s disease.

**Table 4:** Main outcomes regarding the association of circadian rhythm disturbances with MCI

Author	Factor Analyzed	Association with MCI / Dementia
Diem et al. (2016)[16]	Sleep efficiency and latency; sleep variability	Higher risk of MCI/dementia in older women
Gan et al. (2022)[17]	Rotating night shift work (RNSW) and daytime top-up rest (DTR)	RNSW increases the risk of aMCI/dementia; DTR does not compensate for the effects
Tranah et al. (2011)[18]	Circadian rhythms: amplitude, robustness, and phase	Low amplitude/robustness and late acrophase → increased risk
Li et al. (2020)[19]	Circadian rhythm (amplitude, intra/interdaily variability)	Increases the risk of Alzheimer’s in individuals with normal cognition or MCI
Liu et al. (2022)[14]	Nocturnal heart rhythm and oxygen desaturation	Alterations observed in patients with MCI
Naismith et al. (2010)[20]	Awakenings and wake after sleep onset (WASO)	Associated with deficits in attention, learning, and problem-solving
Naismith et al. (2014)[20]	Onset of melatonin secretion, REM, WASO	MCI is related to delayed phase syndrome and poorer memory
Tudela et al. (2014)	Phase advance of temperature, activity, position, light	MCI is associated with an advanced phase in multiple markers
Wams et al. (2017)	Sleep, attention, verbal, and visuospatial memory	Deficits only in verbal/visuospatial memory in aMCI
Yi Lee et al. (2021)[12]	Acrophase (phase of the daily activity rhythm)	Delayed acrophase → lower chance of cognitive improvement

### Studies limitations

The selected studies present several limitations. A recurring issue was the reliance on actigraphy (Diem et al., 2016 [16]; Yi Lee et al., 2021 [12]), which, while objective, cannot capture detailed sleep architecture. Sample homogeneity, particularly the exclusive inclusion of Caucasian women (Diem et al., 2016 [16]; Tranah et al., 2011 [18]) or specific professional groups (Gan et al., 2022 [17]), limits the generalizability of the findings. Small sample sizes were a concern in several studies (Naismith et al., 2010 [13]; Tudela et al., 2014 [15]; Wams et al., 2017 [21]), reducing statistical power and external validity.

Methodological constraints also included the use of self-reported data (Gan et al., 2022) [17], the cross-sectional nature of some designs, which precludes causal inference (Tudela et al., 2014) [15], and short monitoring periods (Tudela et al., 2014 [15]; Yi Lee et al., 2021 [12]). Furthermore, the lack of control for potential confounding variables—such as medical comorbidities, lifestyle habits (Gan et al., 2022) [17], depression (Naismith et al., 2010) [13], antidepressant use (Naismith et al., 2014) [20], and unassessed retinal changes that could affect light input to the suprachiasmatic nucleus (Naismith et al., 2014 [20]) —may have influenced the results. Finally, the absence of in-vivo AD pathology assessment (Li et al., 2020 [19]) and uncontrolled home-environment variables (Wams et al., 2017 [21]) were noted as factors that could impact the interpretation of the association between circadian disruption and cognitive decline.

## Discussion

The joint analysis of the ten studies included in this systematic review points to a significant association between the dysregulation of the circadian system and cognitive decline in the elderly. From a sample of 5,731 participants *across the original study samples (5*,*707 of whom were eligible for analysis under this review’s criteria)* it was possible to verify that changes in circadian rhythm parameters, such as a reduction in their amplitude, stability, and robustness, greater variability, and modifications in their timing (phase), are associated with an increase in the risk of development or progression of mild cognitive impairment (MCI) and dementia in healthy elderly individuals. This correlation extends across three distinct exposure domains — actigraphic rhythm metrics, sleep architecture and lifestyle-based circadian disruption — indicating that the integrity of the circadian system is a multidimensional and relevant factor in the maintenance of cognitive health in elderly populations.

In this study, the 70-year age group was predominant, as was the female population. The mean age of the participants, ranging between 65 and 83 years, reinforces the findings in the literature that relate the advancement of age to greater vulnerability to circadian rhythm disturbances and cognitive impairment. Evidence points out that aging is accompanied by changes in sleep structure and in the synchronization of the biological rhythm, aspects directly linked to the progressive decline of cognitive functions in the elderly population [22–24].

Considering the elderly population segment, longitudinal studies demonstrate that the risk of developing MCI is directly proportional to the advancement of age, especially after 65 years [22, 25, 26]. Similarly, elderly individuals, with or without a diagnosis of dementia, present sleep and circadian rhythm disturbances more frequently compared to younger adults, including difficulty in maintaining regularity in sleep-wake rhythms, in addition to presenting greater sleep fragmentation [27–29]. Furthermore, commonly, aging reduces the circadian rhythm amplitude due to a weakening of rhythmicity, making it difficult for the elderly to adjust to the environmental light cycle, in addition to advancing the sleep phase and decreasing the responsiveness to light [28], as was demonstrated across Domain 1 studies Tranah et al. [18], Li et al. [19]., Liu et al. [14]., and Tudela et al. [15].

A key finding emerging from the cross-domain synthesis is that circadian disruption appears to predict cognitive decline through distinct but convergent mechanisms. Actigraphic studies (Domain 1) demonstrate that reduced rest-activity amplitude and rhythm fragmentation are prospective predictors of MCI and AD progression [12, 18, 19]. Sleep architecture and melatonin studies (Domain 2) reveal that phase misalignment — particularly advanced DLMO — impairs memory consolidation processes that depend on sleep-stage integrity [13, 20]. Lifestyle studies (Domain 3) indicate that environmental light-dark cycle disruption through shift work compounds these risks cumulatively with advancing age [17]. This convergence across methodologically distinct exposure types strengthens the biological plausibility of a circadian-cognition link beyond what any single exposure domain could demonstrate alone.

Recent literature evidence has shown a possible correlation of the risk of developing neurocognitive deficits with circadian cycle changes throughout aging [3, 30]. Notably, two large-scale cohort studies identified during our search further contextualize the findings from Domain 1. Haghayegh et al. [31], leveraging actigraphy data from 91,517 UK Biobank participants followed for up to 7.5 years, demonstrated that suppressed and fragmented rest-activity rhythms — quantified by relative amplitude, intradaily variability, interdaily stability, and acrophase —preceded the onset of MCI and dementia, adjusting for demographics, comorbidities, lifestyle, shift work status, and genetic risk for Alzheimer’s disease [31]. Although the age range of this cohort (43–79 years, with a mean age of 69.6 years among those who developed the outcome) is largely compatible with this review’s age threshold, the study was identified by our original search but was not eligible for inclusion under our pre-defined criteria, because its primary outcome (dementia or MCI) was ascertained through hospital ICD-coded diagnostic records and the UK Biobank’s algorithmically derived dementia date, rather than through the validated cognitive assessment questionnaires required by our protocol. Similarly, Milton et al. (2025) [30], in a five-year prospective cohort of oldest-old women, demonstrated that changes in 24-hour sleep-wake activity patterns were independently associated with incident dementia risk, reinforcing the longitudinal predictive value of rest-activity rhythm metrics documented across the studies included in this review.

Variations in circadian parameters present themselves in distinct ways depending on the MCI subtype and the measurement domain. Studies in Domain 1 (Tranah et al. [18], Li et al. [19]) show that amplitude and robustness reductions prospectively increase the risk of MCI and AD. In the shift work domain, sleep deprivation negatively affected several cognitive functions, including attention, alertness, sensory perception, learning, memory, and executive function (Gan et al. [17]), with effects intensifying with advancing age — consistent with the broader literature on shift work and circadian misalignment [32–35].

A recent population study in China demonstrated that the prevalence of mild cognitive impairment is commonly higher in women (18.9%) when compared to men (10.2%) [36]. However, there is complexity and a dependence on biopsychosocial factors and the MCI subtype regarding the relationship between sex and the risk of developing mild cognitive impairment. A meta-analysis with 56 studies demonstrated a higher prevalence of non-amnestic MCI in women, but not a higher incidence, and there was also no significant distinction between sexes in the context of the other subtypes of mild cognitive impairment [37]. In this review, the female sex prevailed in the sample, with 5,307 participants (92.6% *of the 5*,*731 original study enrollment figure*,* calculated on the same denominator as the headline total reported above*). The study by Liu et al. [14]., also based in China, demonstrated that, despite the slightly higher number of female participants, the higher incidence of MCI in this group (71.6%) than in the male sex (64.1%). In contrast, other studies show that the incidence of MCI, in both the amnestic and non-amnestic subtype, can be higher in the male sex than in the female sex [38, 39], as occurred in Naismith et al., with men representing 65% of the MCI group, in a scenario of only six patients differing from the total of participants by sex.

Three of the ten included studies were conducted in the United States; however, the systematic review also covered research originating from Asia, Europe, and Oceania, evidencing the wide geographic dispersion and population diversity of the studies on the theme. A meta-analysis with 66 studies and more than 200 thousand participants aged 50 years or older identified an MCI prevalence of about 15% (95%CI: 13.24–18.03%) throughout the world, considering factors such as age, schooling, gender, and region of influence. The amnestic subtype presented 10.03% (95%CI: 7.98–12.27%) prevalence and the non-amnestic 8.72% (95%CI: 6.78–10.89%), in line with the results of this review, in which there was a predominance of the first subtype.

Wams et al. [21] was the only study to present a distinct outcome: despite a reduction in verbal and visuospatial memory capacity, participants with aMCI did not diverge from controls in sleep quality, quantity, or circadian amplitude. This finding is particularly relevant because it suggests that, in certain MCI subtypes, cognitive impairment may emerge prior to measurable circadian amplitude changes — implying that amplitude metrics alone may be insufficient as early biomarkers in all subtypes. Future research should consider combining amplitude with phase and variability metrics for more sensitive detection.

### Limitations

The population restriction to the female sex in Diem et al. [16], Gan et al. [17], and Tranah et al. [18], with the first and third using the same database of study centers, and the prevalence of male sex in only three of the ten studies, are some of the limitations of this work. The same occurs due to the small sample in 50% of the studies and the large database in the others, in addition to the collections carried out in outpatient and hospital centers by Gan et al. [17], Naismith et al. [20], Naismith et al. [13, 20], and Wams et al. [21], and the non-specification of the mean age of the subgroups in three articles (Table 1). Three studies, Gan et al. [17], Liu et al. [14], and Tudela et al. [15], did not provide exact numerical data on objective sleep patterns (Table 3), which hinders a more precise and direct analysis of circadian variations. The distinct study designs, with different follow-ups and analyses in the target population, in addition to the varied tools for assessing cognitive health and sleep-wake parameters, impact the specificity of the results in this review, for example, the total prevalence of MCI in patients with circadian rhythm disturbances in the population covered by heterogeneous selection and follow-up methods. Finally, although the search strategy was re-run in June 2026 and did not identify additional eligible studies, the original search was not formally re-executed with an extended end date and re-screened beyond this verification step; consequently, evidence published very close to the submission date may not be fully captured, and this is acknowledged as a limitation of the review.

## Conclusion

The results of the present systematic review evidence that changes in sleep quality, directly influenced by circadian sleep-wake patterns and lifestyle, negatively impact cognitive health during aging. This review’s three-domain framework — encompassing actigraphic rest-activity metrics, sleep architecture and lifestyle-based circadian disruption — provides a structured synthesis of the available evidence that extends beyond prior reviews focused on individual exposure categories. However, the literature lacks in-depth studies on the incidence and consequences of circadian rhythm disturbances in the elderly population in the world. From this perspective, these findings reinforce the importance of future works investigating more specifically and in detail the existence of a physiological association between circadian rhythm disturbances and the diagnosis of mild cognitive impairment, analyzing the impact that they can have on the quality of life of patients and on healthy aging.

## Data Availability

All data generated or analyzed during this study are included in this published article.
